# DoE‐It‐Yourself (DoEIY): An Open‐Access Web Application for Democratizing Experimental Design in Chemical and Materials Research

**DOI:** 10.1002/smtd.202501779

**Published:** 2026-01-21

**Authors:** Niamh Mac Fhionnlaoich, Ye Yang, Runzhang Qi, Federico Galvanin, Stefan Guldin

**Affiliations:** ^1^ University College London, Department of Chemical Engineering London UK; ^2^ APC Ltd. Dublin Ireland; ^3^ Langmu Bio Yuhang Hangzhou China; ^4^ Technical University of Munich, Department of Life Science Engineering Freising Germany; ^5^ TUMCREATE Ltd Singapore Singapore

**Keywords:** DoE, design of experiments, data‐driven experimentation, experimental design software, open‐access tools

## Abstract

Achieving predictable and reproducible outcomes is a central challenge across synthetic workflows. Design of Experiments (DoE) offers a structured, multivariate framework for exploring complex parameter spaces, yet its wider adoption has been limited by statistical complexity, licensing costs, and steep learning curves. To address these barriers, we introduce DoEIY.app, an open‐access web application that streamlines the experimental design process. The software supports guided design generation, data entry and analysis, and interactive model exploration through an intuitive interface. We illustrate its use in two case studies on gold nanoparticle synthesis, focusing on minimizing size dispersity and controlling mean particle diameter. These examples demonstrate how DoEIY.app enables efficient, reproducible process optimization and highlight its potential to democratize DoE implementation across a broad range of scientific domains.

## Graphical abstract

Reproducible and efficient experimental workflows remain a persistent challenge in chemical and materials research. Design of Experiments (DoE) provides a powerful multivariate framework but is often limited by complexity and accessibility. DoEIY.app is a free, browser‐based web application that enables guided experimental design, analysis, and interactive model exploration, demonstrated through gold nanoparticle synthesis case studies.

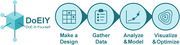

## 1 Introduction

Planning and executing experimental workflows is central to scientific discovery, yet achieving reproducible and efficient outcomes remains a persistent challenge across disciplines. A common approach is to vary one factor at a time (OFAT) while holding all others constant. While intuitive, OFAT provides only a limited view of system behavior, fails to capture interactions between variables, and often leads to suboptimal or poorly reproducible results [1]. In contrast, Design of Experiments (DoE) provides a structured, multivariate framework for process development and optimization. By systematically varying multiple factors, DoE reduces the influence of experimental noise, enables detection and quantification of interactions, and yields deeper insight into complex systems [2, 3]. These features make DoE a significantly more powerful and informative strategy for planning and executing experimental workflows.

Besides applications in polymer synthesis, biomaterials, food systems, pharmaceuticals, and many other domains, the synthesis of nanoparticles exemplifies both the need for and the potential of such approaches. Their importance in catalysis, biomedicine, sensing, and photonics arises directly from their well‐known size‐dependent properties such as surface reactivity and optical response [4, 5, 6, 7]. Yet, achieving consistent control over particle size and dispersity has long been recognised as difficult. Because nucleation and growth are complex and strongly influenced by experimental conditions, simple one‐factor‐at‐a‐time methods are insufficient for ensuring reproducibility.

Despite the clear advantages, the routine use of DoE in nanoparticle synthesis remains relatively uncommon. A recent review of publications on nanomedicine indexed in PubMed from 2000 to 2023 reported that only 2% of the studies applied DoE methodologies [8]. This trend extends beyond nanomedicine to other nanosciences and indicates a significant gap between statistical best practices and everyday experimental workflows. When DoE has been applied, it has shown significant benefits. For instance, Hao et al. optimized solid lipid nanoparticles for drug delivery and revealed strong factor interactions that would have been missed using OFAT methods [9]. Barglik‐Chory et al. used a response surface design to tune the band gap energies of bio‐stabilized CdS nanoparticles, highlighting key non‐linear effects [10]. Similarly, Burrows et al. applied a fractional factorial design to efficiently study eight variables in the seed‐mediated silver‐assisted synthesis of gold nanorods, uncovering new process insights [11]. Numerous other studies have successfully leveraged DoE to optimize nanoparticle characteristics such as size, phase, stoichiometry, yield, and drug loading [12, 13, 14, 15, 16, 17, 18, 19, 20, 21, 22, 23, 24, 25].

The limited uptake in synthetic workflows likely stems from multiple causes; however, there are several key barriers. First, DoE involves some statistical complexity, requiring an understanding of various experimental designs, model selection, analysis, and interpretation. Second, accessibility remains a significant hurdle. Widely used DoE platforms like JMP or Design Expert require a commercial license. Additionally, their extensive and advanced features often come with a steep learning curve. Third, while open‐source alternatives, such as DoE.base in R, offer powerful, no‐cost solutions, they lack user‐friendly interfaces, deterring those without programming experience. An additional barrier is the limited coverage of DoE in most undergraduate and postgraduate science programmes, meaning that many researchers enter the laboratory with little prior exposure to structured experimental design [26].

Recognizing these challenges, the scientific community has made notable contributions to bridge this gap. For example, Murillo et al. developed FielDHub, a free, user‐friendly Rshiny app specialized in DoE for life sciences [27]. Williamson et al. published a seminal how‐to guide for applying DoE to nanocrystal synthesis, outlining best practices from design planning through analysis [28]. Still, there remains no general‐purpose, open‐access tool that can support the needs of experimental scientists across diverse fields.

To close this gap, we introduce DoEIY.app. a lightweight web application based on RShiny, that provides a guided DoE workflow without the need for specialized software or coding skills. DoEIY offers a practical and flexible suite of experimental design tools, supporting both classical designs—such as full and fractional factorial, Plackett–Burman, Box–Behnken, and Central Composite—as well as non‐classical or computer‐generated designs, including Latin Hypercube and D‐optimal. This breadth enables users to tailor their design strategies to different levels of complexity, resource availability, or data requirements. In addition, the software supports intuitive model building, design diagnostics, and interactive exploration of experimental responses. Freely accessible via the web, DoEIY.app is designed to accommodate a broad range of experimental workflows across scientific domains. We demonstrate the utility of this tool through two case studies in gold nanoparticle (AuNP) synthesis: (1) minimizing dispersity and (2) tuning particle size. The DoE workflow implemented in the web application made it possible to resolve factor interactions and account for experimental variability, which in turn enabled reproducible synthesis of monodisperse nanoparticles with controlled mean diameters.

## 2 Methods

### 2.1 DoEIY Software

**FIGURE 1:**
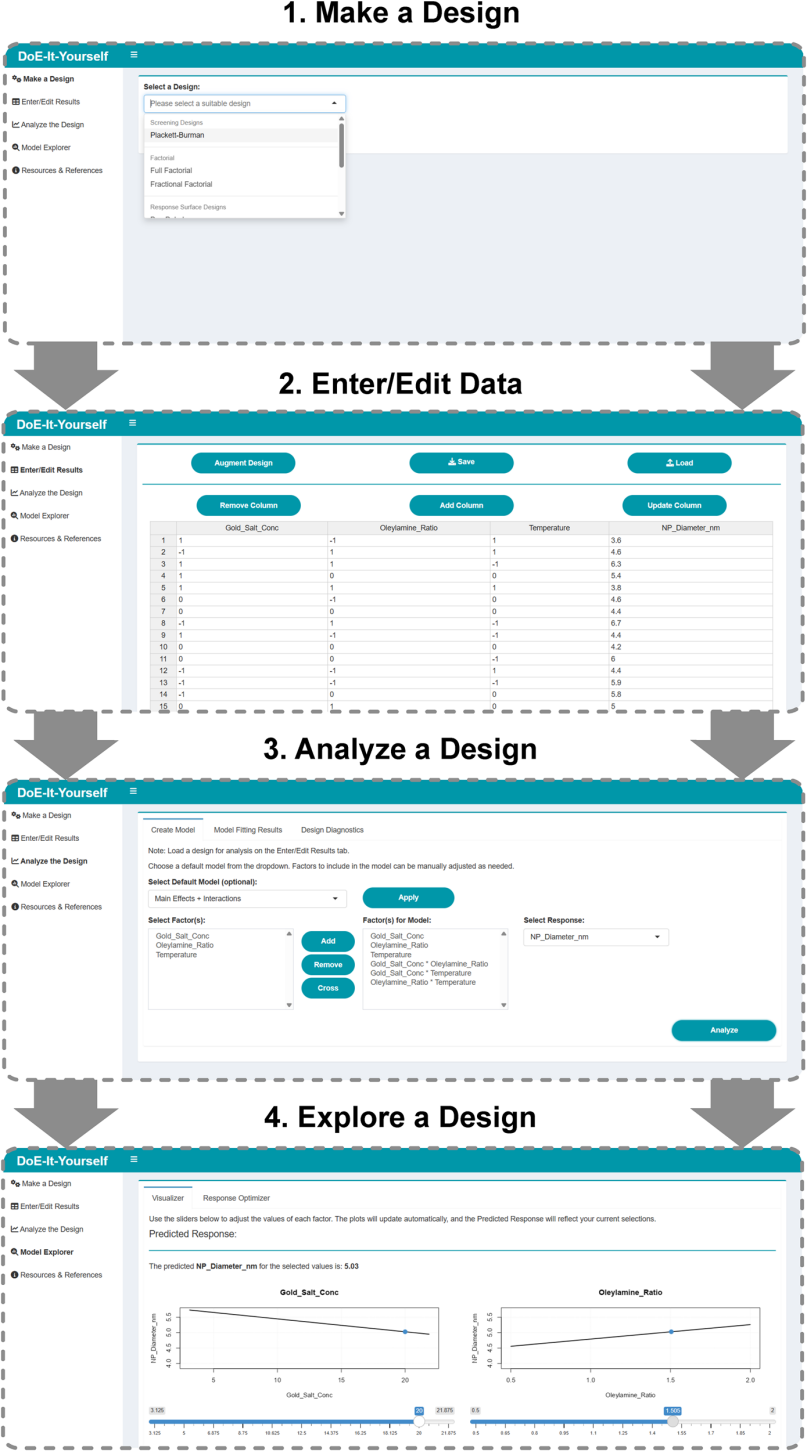
Overview of the DoEIY software workflow. 1. Make a Design: users select a design type, define factors, and generate the experimental design. 2. Enter/Edit Results: experimental responses are entered directly into the editable design table, with options to save or load designs. 3. Analyze the Design: users select or create custom models; the model summary and statistics as well as design diagnostics are presented. 4. Explore the Design: interactive visualization of factor–response relationships and optimization and prediction tools allow users to identify factor settings that maximize, minimize, or achieve a target response.

DoEIY.app, our DOE software, was built using R (v4.4.1) and Rshiny (1.10.0). This browser‐based web application was designed to guide the user through the complete DoE workflow, from experimental design to data entry, to analysis and optimization. An overview of the software is shown in Figure 1.

The **Make a Design** module serves to guide the user through generation of experimental designs. A range of designs is provided to accommodate common experimental scenarios, such as screening and response surface methodologies. The available designs are summarized in Table 1.

**TABLE 1:** Design Generation Methods. Overview of the available experimental designs, their classification and implementation details, including underlying algorithms, source publications and associated R packages (where applicable).

Design	Type	Methodology
**Plackett–Burman**	Screening	Constructed using generators described in the original publication for 4‐23 factors [29].
**Full Factorial**	Factorial	Generated using the expand.grid() base R function. Available for up to 25 factors.
**Fractional Factorial**	Factorial	Generated using the FrF2 R package (v2.33) for 3‐25 factors [30].
**Box–Behnken**	Response Surface	Constructed using matrices described in the original publication for 3‐7 factors [31].
**Central Composite**	Response Surface	Generated using the rsm R package (v2.10.5) for 2 or more factors [32].
**Latin Hypercube**	Space Filling	Generated using the lhs R package (v1.2.0) for any number of factors [33].
**D‐Optimal**	Custom	Generated using the AlgDesign R package (v1.2.1.1) for any number of factors [34].

For each type of design, the software imposes the necessary constraints (e.g., allowable factor types, permissible number of levels) and prompts the user to define factor details. Depending on the design, users may also select blocking options and specify the number of experimental runs. Once these parameters are specified, the software generates the design using the corresponding methodology outlined in Table 1.

Once a design has been created, the **Enter/Edit Results** section presents it as an editable table allowing users to enter experimental results directly. Designs can be saved for future use or reloaded for further work. Additionally, columns and rows can be added, removed, or modified in this interface.

To support data traceability, a metadata column is automatically included when a new design is created. Users may use this field to record information relevant to reproducibility—such as experimental details, reagent batch numbers, or instrument identifiers—and can add further metadata columns as required.

To accommodate situations where additional influential factors are identified during experimentation, DoEIY now includes a beta version of a design‐augmentation feature. Through the “Augment Design” option, users can modify or add factors and generate a D‐optimal augmentation based on the runs already completed. This enables refinement of the experimental design without needing to reconstruct the study from the beginning.

The **Analyze the Design** module provides model fitting and statistical evaluation. Users can select a default model structure (main effects only, main effects plus two‐way interactions, or a response surface model) or build a custom model by adding, removing, or crossing factors to form interaction terms. The model is then fitted using a least‐squared regression via the aov() function and an analysis of variance (ANOVA) is applied to evaluate the model significance. The results of this include a summary of the model statistics (R^2^, adjusted R^2^, F‐statistic with degrees of freedom, and p‐value) and a parity plot comparing the predicted vs. experimental values. The ANOVA table lists each model term with the associated degrees of freedom, sum of squares, mean square, F‐statistic, and p‐value, with statistical significance indicated by asterisks. The Estimates table provides the coefficients for the factors alongside their standard error and t‐statistic.

Post model fitting, design diagnostics are available to evaluate the design's effectiveness. Where applicable, it displays the alias structure, which identifies model terms that are indistinguishable from one another due to the experimental design. A correlation heatmap is also provided to assess collinearity between model terms, helping determine whether the effects can be estimated independently.

The **Model Explorer** tab presents interactive factor‐response plots with sliders to adjust the factor levels dynamically; changes in one factor automatically update dependent factor plots for models with interaction effects. It also presents the predicted response for the given factor settings. Additionally, this tab includes a module for model‐based optimization, allowing users to define a goal (maximization, minimization, or achieving a target value) and optionally supply starting values. The optimizer returns the factor settings predicted to meet the goal along with the corresponding predicted response. Optimization is performed using the optim function from the base R stats package, employing the L‐BFGS‐B method for bound‐constrained optimization [35].

DoEIY enables users to design experiments, record results, perform statistical analysis, visualize factor effects, and conduct optimization within a single, easy‐to‐use workflow. Detailed guidelines are provided in the supplementary user guide.

The case studies that follow are intended solely as illustrative demonstrations of the workflow; the DoEIY architecture is domain‐agnostic, and users may define their own experimental factors and responses for application across a wide range of problems in chemical and materials research.

### 2.2 Synthesis

The general protocol to synthesis oleylamine‐capped AuNPs required the gold precursor ${\mathrm{HAuCl}}_{4}\cdot 3H_{2}O$ to be dissolved in 20 mL mixture of octane and oleylamine, the ratio of which was set according to the experimental design. A jacketed flask was connected to a Grant GD120‐R2 thermostatic bath which maintained a set temperature with a resolution of 0.1 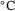. The flask was flushed with argon to provide an inert atmosphere then sealed. The gold precursor solution was stirred vigorously for 10 min to ensure full dissolution and to equilibrate the temperature in the flask. A second solution was prepared containing tert‐butylamine borane (tBAB) in octane and oleylamine at a concentration of 0.125 M. Once fully dissolved, this solution was quickly injected into the jacketed flask containing the gold precursor. After a set reaction time (30 min to 2 h), the reaction was quenched with ethanol. The quantities of ${\mathrm{HAuCl}}_{4}\cdot 3H_{2}O$, molar equivalents of tBAB, temperature, and composition of the octane/oleylamine solution were determined by the experimental design. The ranges for the experimental conditions are summarized in Tables 2 and 3.

**TABLE 2:** Experimental factors for nanoparticle dispersity. Factors included in the DoE study, with values corresponding to the low, medium, and high levels.

Factor	−1	0	+1
Reducing Agent Stoichiometric Ratio	0.5	1.25	2
Reaction Time (minutes)	30	75	120

**TABLE 3:** Experimental factors for mean nanoparticle diameter. Factors included in the DoE study, with values corresponding to the low, medium, and high levels.

Factor	−1	0	+1
Gold precursor Concentration (mM)	3.125	12.5	21.875
Ratio of Oleylamine to Reaction Solvent	0.5	1.25	2
Reaction Temperature ( 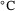 )	5	15	25

To wash the particles, the reaction solution was divided between two 50 mL Eppendorf tubes and precipitated using ethanol. A Thermo Scientific Multifuge X1R was used to centrifuge the samples at 5000 rpm and 10 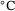 for 10 min. After decanting, approximately 2 mL dichloromethane was used to resuspend the particles before the washing protocol was repeated. The AuNPs were left to dry overnight under vacuum at room temperature. TEM images were obtained using a JEOL JEM‐2100 (200 kV). The images were analysed by imageJ to determine the area of the AuNPs and, assuming spherical particles, the diameter of each NP was obtained. The resolution of the measurement for the image acquisition and analysis was estimated to be 0.1 nm. This was used as the bin width to calculate the nanoparticle entropy [36].

### 2.3 Design of Experiments

Two designs were used in the study of these oleylamine‐capped AuNPs. The first experimental campaign was used to determine a set of conditions which minimized the dispersity of the resulting nanoparticle population. The aim of the second design was to investigate the effect of experimental conditions on the mean particle size and develop a model. These two designs are detailed below.

#### 2.3.1 Case Study 1: Minimizing Nanoparticle Dispersity

A three level, two factor full factorial design was used to study the effects of the reaction time and ratio of the reducing agent to the gold precursor on the nanoparticle dispersity. The center point was replicated three times to assess experimental variation. Previous studies on this and similar systems had highlighted these two as key variables in the dispersity of the resulting population and this design would provide a response surface model to reveal any non‐linear effects or interactions between the two factors. The levels investigated are presented in Table 2. For these experiments, a solution of 12.5 mM of gold precursor in solution of 6.67 mL of oleylamine and 13.33 mL of octane was prepared. The reaction temperature was set at 15 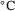. The full experimental design can be found in Supporting Information Table 1.

#### 2.3.2 Case Study 2: Modeling Nanoparticle Diameter

Prior work on the synthesis of oleylamine‐capped AuNPs has identified the concentration of the gold precursor and capping agent as well as the reaction temperature as experimental variables by which mean particle diameter could be controlled. Here, a central composite inscribed design was used to explore the full experimental domain and develop a response surface model for mean nanoparticle diameter. This experimental design is shown in Supporting Information Table 2. The values corresponding to the low, medium, and high levels are summarized in Table 3. A stoichiometric ratio of 1.6 moles of tBAB to gold precursor and a reaction time of 120 min was used for this design.

## 3 Results and Discussion

The performance and utility of DoEIY was evaluated through two case studies in AuNP synthesis. The first focused on minimizing particle dispersity, while the second examined how to tune particle size. These studies highlight typical challenges encountered in nanomaterials process development and demonstrate how DoE can reveal important interactions and provide meaningful insights even for noisy systems. Rather than applying a single design to study all factors simultaneously, the experimental campaign was divided based on prior knowledge from literature that distinct parameters govern dispersity and particle size. Furthermore, in highly polydisperse systems, it is difficult to estimate a meaningful measure of mean particle diameter, particularly when multiple size populations are present within a single sample. By first identifying and optimizing the conditions that minimized dispersity, a controlled baseline could be established to enable effective modelling of particle diameter in the subsequent experimental campaign.

### 3.1 Case Study 1: Dispersity

To investigate the impact of synthesis conditions on nanoparticle dispersity, a three‐level full factorial design was implemented using the **Make a Design** module of the DoEIY web application. This allowed the estimation of both second‐order effects and interactions between the two critical factors, the stoichiometric ratio of reducing agent to gold precursor and the duration of the reaction. A modified form of information entropy was used to quantify the dispersity of the nanoparticle populations [36]. While commonly used metrics such as polydispersity index (PDI) or standard deviation describe width or spread, they can be sensitive to skewed or multimodal distributions and may fail to fully capture population heterogeneity. Entropy offers a more general statistical measure of disorder in the size distribution, capturing the complete shape of the histogram without requiring assumptions about modality or symmetry. A response surface model was used to fit the data, including main effects, two‐factor interactions, and quadratic terms. The results of the fitted model are summarized in Table 4.

**TABLE 4:** Experimental factors determining dispersity. Model terms, scaled parameter estimates, and associated p‐values for nanoparticle dispersity as quantified by entropy.

Factor	Scaled Estimate	p‐value
Intercept	1.6153	0.0002
Reducing Agent Stoichiometric Ratio	−0.2383	0.1183
Reaction Duration (minutes)	−0.5683	0.0065
[Reducing Agent Stoichiometric Ratio] 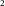	0.5718	0.0324
Reducing Agent Stoichiometric Ratio]* [Reaction Duration (minutes)]	−0.3500	0.0735
[Reaction Duration (minutes)] 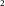	0.4518	0.0735

Model fitting was performed within the **Analyze the Design** module using a response surface model. The ANOVA table, parameter estimates, and model statistics (R^2^, adjusted R^2^, F‐statistic, and p‐values) were automatically generated by the application and key results are shown in Table 4. A parity plot comparing predicted versus observed responses provided additional visual confirmation of model fit. Furthermore, the **Design Diagnostics** tab indicated that no model terms were aliased, supporting that the factorial design provided sufficient resolution to estimate all specified main effects, interactions, and quadratics independently. A full overview of this process, from making the design through to the model exploration is summarized in Supporting Information Figure 1.

The model exhibited strong predictive performance, with an RMSE of 0.31 and R^2^ of 0.91 (adjusted‐R^2^ = 0.81), and a statistically significant overall fit (model p‐value = 0.013). It was clear that the interaction and second‐order terms were particularly important in describing the system's behavior. These insights would not have been revealed through an OFAT approach, which inherently cannot capture interactions between factors. The **Model Explorer** module was then used to visualize how the reducing agent ratio and reaction time influence dispersity. This module also provides functionality to maximize, minimize, or target a response value based on the fitted model. Using this feature, the entropy was minimized at a reducing agent ratio of 1.6 and a reaction time of 111 min, corresponding to the lowest predicted entropy (i.e. dispersity).

**FIGURE 2:**
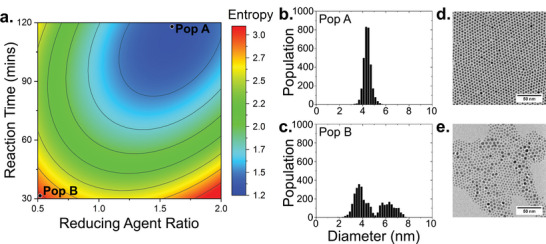
Dispersity model. (a) Response surface of reducing agent ratio and reaction time on nanoparticle dispersity (entropy metric). (b,c) AuNP diameter distributions at experimental conditions A and B, respectively. (d,e) Corresponding TEM images of samples A and B.

While DoE models are powerful tools for uncovering trends and interactions, they typically assume linear or quadratic relationships within the defined design space. This assumption enables efficient exploration and model fitting, but it can also oversimplify the underlying system behavior, particularly in complex, non‐linear processes. As such, interpretation of the model output must be tempered with domain knowledge. Oleylamine is a well‐established and efficient capping agent, and literature suggests it provides robust stabilization against aggregation, which would lead to an increase in dispersity. This is supported by the experimental data, which shows that dispersity decreases with increasing reaction time and then plateaus. The effects of reducing agent ratio and reaction time on the entropy are shown in Figure 2.

These findings align well with previous studies on AuNP synthesis. Prior studies have established that gold nanoparticle synthesis involves an initial nucleation phase followed by a slower growth phase, during which coalescence and Ostwald ripening lead to reduced dispersity [37, 38, 39]. In this context, premature interruption of the reaction can result in polydisperse populations, while sufficient reaction time allows the system to equilibrate toward uniform particle sizes. Liu et al. further demonstrated that this size‐focusing over time followed a logistic trend, consistent with the asymptotic dispersity behavior observed here [39].

The role of the reducing agent was similarly nuanced. Zheng et al. reported that strong or highly concentrated reducing agents can lead to rapid reduction and uncontrolled nucleation, resulting in high dispersity [40]. In contrast, weaker or more dilute reducing agents promoted a slow, controlled nucleation and growth, reducing dispersity. However, insufficient reducing agent was also noted to potentially generate highly disperse populations [41, 42]. The model in this work identified a significant quadratic relationship between reducing agent ratio and entropy, suggesting that both too much or too little reducing agent results in suboptimal outcomes.

### 3.2 Case Study 2: Mean Diameter

To accurately determine the factors that affect the mean particle diameter, monodisperse, or near‐monodisperse, populations are required. In highly polydisperse or multimodal systems, any estimate of average diameter becomes unreliable, obscuring the underlying trends and limiting interpretability. Therefore, the optimized conditions identified in Case Study 1 for minimizing dispersity were applied across all runs in this second design.

Using DoEIY's **Make a Design** module, a face‐centered central composite design (CCD) was created to investigate the influence of three continuous variables: gold precursor concentration, the molar ratio of oleylamine to reaction solvent, and reaction temperature. This response surface design enabled a comprehensive evaluation of the design space by capturing main effects, quadratic terms, and two‐way interactions. The resulting design was displayed in the **Enter/Edit Data** tab. where the experimental results were added for analysis.

The dataset was then analyzed in the **Analyze the Design** module, where a response surface model was fitted. Insignificant effects were iteratively removed until only statistically significant contributors to the response remained. Results of the final, fitted model are shown in Table 5, and the software provided accompanying statistical summaries including model R^2^, adjusted R^2^, RMSE, ANOVA tables, and parameter estimates. A complete summary of this DoE process is presented in Supporting Information Figure 2.

**TABLE 5:** Experimental factors determining mean gold nanoparticle diameter. Model terms, scaled parameter estimates, and associated p‐values for mean AuNP diameter.

Factor	Scaled Estimate	p‐value
Intercept	4.9625	<0.0001
Gold precursor Concentration (mM)	−0.3900	0.0287
Ratio of Oleylamine to Reaction Solvent	0.3500	0.0456
Reaction Temperature ( 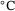 )	−0.8600	0.0001

**FIGURE 3:**
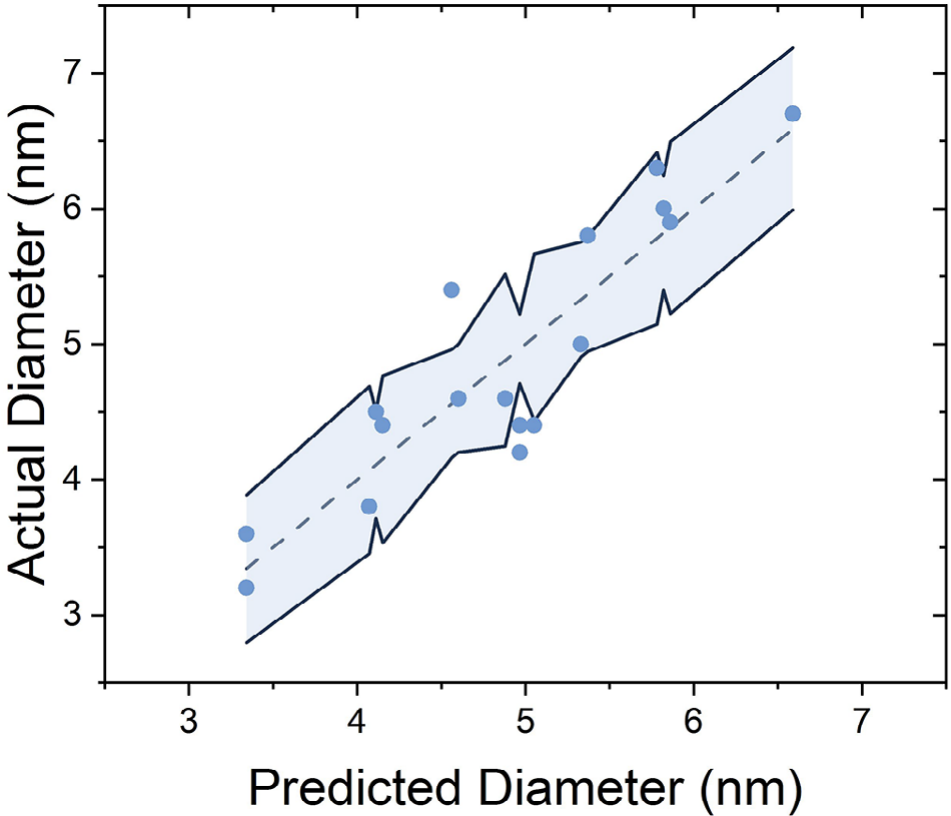
Actual vs. predicted mean particle diameter. Measured particle diameters plotted against values predicted by the fitted response surface function.

These goodness of fit metrics suggested the model satisfactorily captured the underlying trends in the data, with an R^2^ of 0.77 and an RMSE of 0.50 (adjusted R^2^ = 0.72), and a statistically significant fit (p‐value = 0.0003). These values reflect good agreement between the predicted and experimental results; Figure 3 plots the actual data against the predicted values.

**FIGURE 4:**
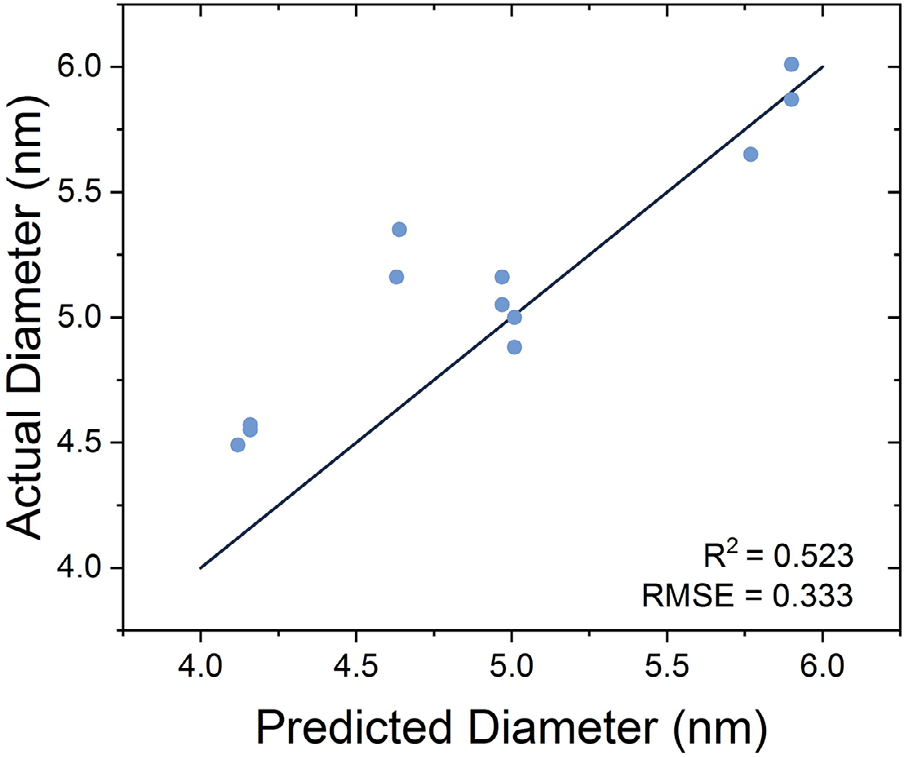
Gold nanoparticle mean diameter model validation. Actual data plotted against the predicted values for the mean particle diameter model.

To validate the predictive capabilities of the model, a series of additional experiments were conducted targeting AuNP synthesis in the 4–6 nm range. The required experimental conditions were identified using the model from Table 5. The experimental results aligned well with the predicted values, yielding an R^2^ of 0.52 and RMSE of 0.33 (Figure 4), confirming the model's utility despite the experimental variance.

**FIGURE 5:**
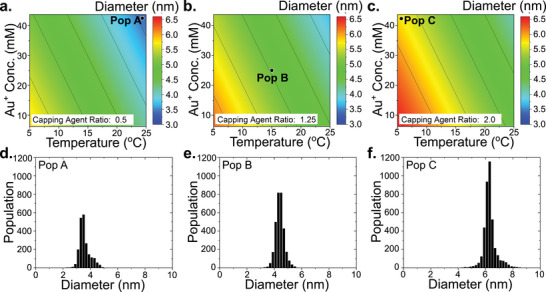
Effect of experimental conditions on mean gold nanoparticle diameter. Response surfaces showing the combined effects of gold precursor concentration and reaction temperature at fixed oleylamine‐to‐octane rations of (a) 0.5 (low level), (b) 1.25 (centre point), and (c) 2.0 (high level). Corresponding AuNP diameter distributions are plotted in (d–f) with reference to the corresponding experimental conditions.

All three factors, gold precursor concentration, reaction temperature, and composition of the solvent solution, were important in governing the mean particle size. The relationship between these parameters is shown in Figure 5. The strong effect of reaction temperature on mean particle size has been well established by previous studies [43, 44, 45]. In this study, temperature emerged as the dominant factor, with higher temperatures yielding smaller particles.

The ratio of oleylamine to solvent also significantly influenced particle size. The efficacy of oleylamine in forming a stabilising layer on the surface of the nanoparticle depends on the temperature and relative concentration of oleylamine [39, 46]. In agreement with the work done by Aslam et al., a decrease in the concentration of the oleylamine corresponds to an increase in particle size.

Additionally, it was found that the particle diameter varied proportionally with the concentration of the gold precursor in the range investigated. This dependence has been noted in the synthesis of citrate and thiol stabilized AuNPs [41, 47, 48]. It is difficult to make direct comparisons with such different systems; however, these lend credence to the impact of gold precursor on the mean particle size. Furthermore, the profoundly non‐linear behavior observed by Zabetakis et al. on the effect of the ratio between the citrate and the gold precursor and the mean particle size cautions against extrapolation of this effect to other concentrations.

This case study further underscores the importance of DoE in systems subject to experimental variability. The observed variation in measured outcomes, if approached via OFAT, could easily obscure key factor effects or produce misleading trends. In contrast, DoE compares group means rather than individual measurements, allowing underlying trends to be identified even in the presence of experimental noise. Recent advances in AI‐guided experimentation and self‐driving laboratories highlight the promise of active learning and closed‐loop control in chemical synthesis [49, 50, 51]. While such approaches enable predictability, they often rely on large and diverse datasets, specialized infrastructure, and complex integration layers. Furthermore, they may obscure the direct link between input variables and experimental outcomes. In this context, classical DoE and AI‐guided approaches are best viewed as complementary, with DoE offering a robust and interpretable framework that can provide structured, low‐noise datasets as foundation for subsequent AI‐guided closed‐loop experimentation.

## 4 Conclusion

Design of Experiments (DoE) provides a powerful framework for understanding and optimizing synthetic workflows, yet its uptake has remained limited due to accessibility and usability challenges. The DoEIY application addresses these barriers by offering a freely available web application that makes DoE user‐friendly and broadly accessible.Through two case studies in AuNP synthesis, DoEIY proved effective even in systems characterized by process noise and subtle factor interactions. These examples highlight the critical role of DoE not only in optimization, but in deepening process understanding. Overall, DoEIY empowers researchers to incorporate DoE into everyday experimentation, advancing reproducibility and efficiency across nanoscience, chemistry, life sciences, and materials engineering.

## Conflicts of Interest

The authors declare no conflict of interest.

## Supporting information




**Supporting File**: smtd70443‐sup‐0001‐SuppMat.pdf

## Data Availability

The data that support the findings of this study are available in the supplementary material of this article.
