# Supplementary material for: DoE‐It‐Yourself (DoEIY): An Open‐Access Web Application for Democratizing Experimental Design in Chemical and Materials Research
Source: Small Methods. 2026 Jan 21;10(4):e01779. doi: 10.1002/smtd.202501779 (PMC12929928; doi:10.1002/smtd.202501779)
Supplement: Supplementary file 1 — Supporting File: smtd70443‐sup‐0001‐SuppMat.pdf [file SMTD-10-e01779-s001.pdf]

# – SUPPORTING INFORMATION –

DoE-It-Yourself (DoEIY): An Open-Access Web Platform for Democratizing Experimental Design in Chemical and Materials Research

Niamh Mac Fhionnlaoich,[*,†,‡] Ye Yang,[†,¶] Runzhang Qi,[†,¶] Federico Galvanin,[†] and Stefan Guldin[*,†,§,‖]

†*University College London, Department of Chemical Engineering, London, WC1E 7JE, United Kingdom*

‡*APC Ltd., Dublin, D18 DH50, Ireland*

¶*Langmu Bio, Yuhang, Hangzhou 311112, China*

§*Technical University of Munich, Department of Life Science Engineering, 85354 Freising, Germany*

‖*TUMCREATE Ltd, Singapore, 138602, Singapore*

E-mail: nmacfhionnlaoich@gmail.com; guldin@tum.de

Case Study 1: Minimizing Nanoparticle Dispersity

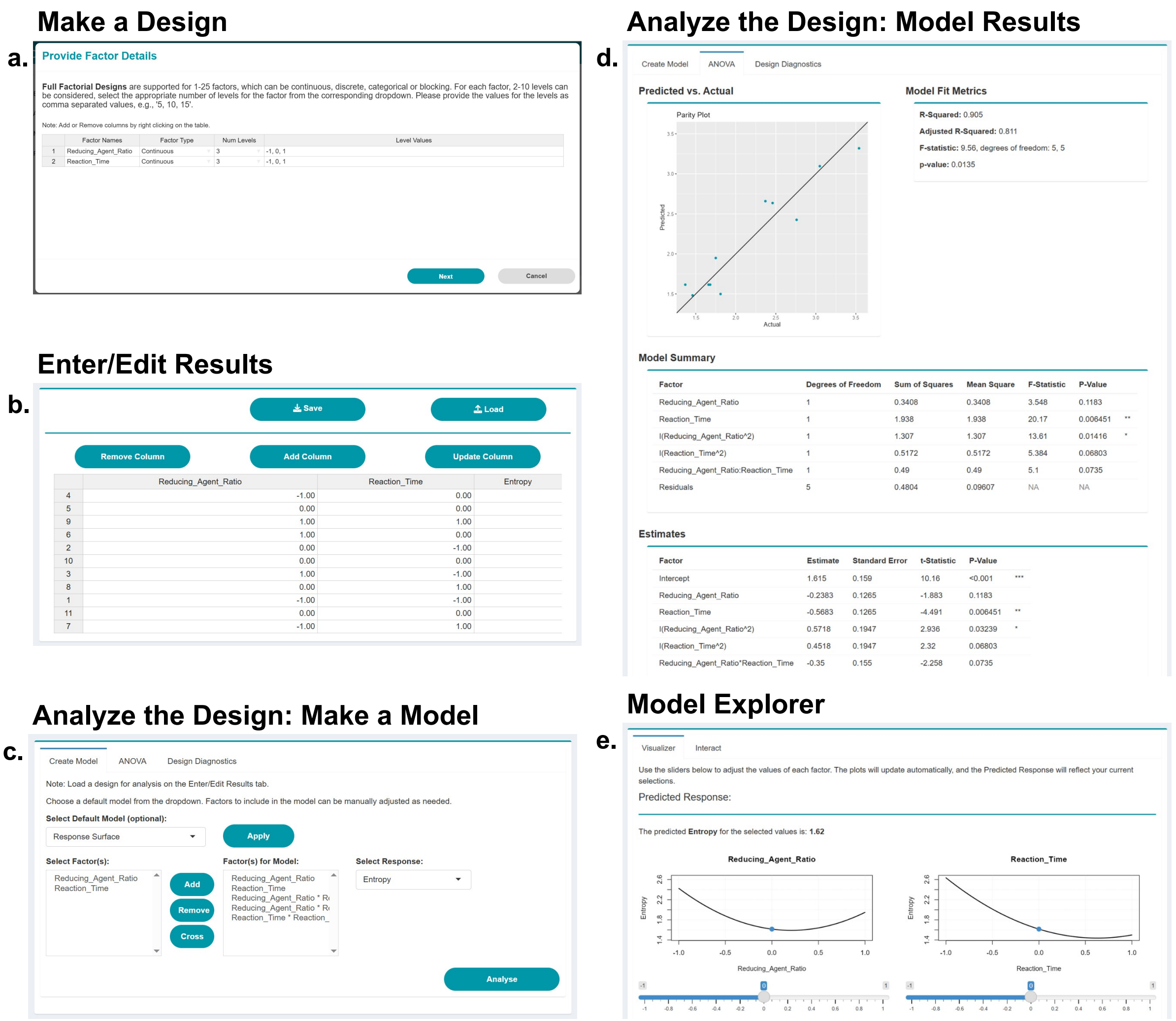

Figure S1: **Case Study 1: Full factorial design workflow.** Workflow for Case Study 1 demonstrating use of DoEIY. **a.** Creation of a three-level full factorial design using the **Make a Design** module. **b.** The design displayed in the **Enter/Edit Data** tab for entry of experimental results. **c.** Model specification and analysis using a response surface model within the **Analyze the Design** module. **d.** Model fitting results, including parity plot, goodness-of-fit statistics, ANOVA, and parameter estimates. **e.** Exploration of factor effects on the response using the **Model Explorer**.

Table S1: **Full experimental design for Case Study 1: Nanoparticle Dispersity** The 3-level, full factorial design used to investigate the role of the stoichiometric ratio of reducing agent to gold salt and the reaction duration on the nanoparticle dispersity as measured by the nanoparticle entropy.

**Run**	**Reducing Agent Stoichiometric Ratio**	**Reaction Duration (minutes)**	**Diameter (nm)**	E	E_n
1	0	0	3.8	1.37	0.36
2	+1	-1	4.9	3.54	0.72
3	-1	-1	4.7	3.05	0.64
4	-1	0	4.5	2.76	0.62
5	0	-1	3.8	2.46	0.64
6	+1	0	3.9	1.75	0.44
7	+1	+1	4.0	1.46	0.37
8	0	0	4.1	1.66	0.40
9	-1	+1	4.7	2.37	0.51
10	0	+1	4.3	1.81	0.42
11	0	0	4.4	1.68	0.38

Case Study 2: Modelling Nanoparticle Diameter

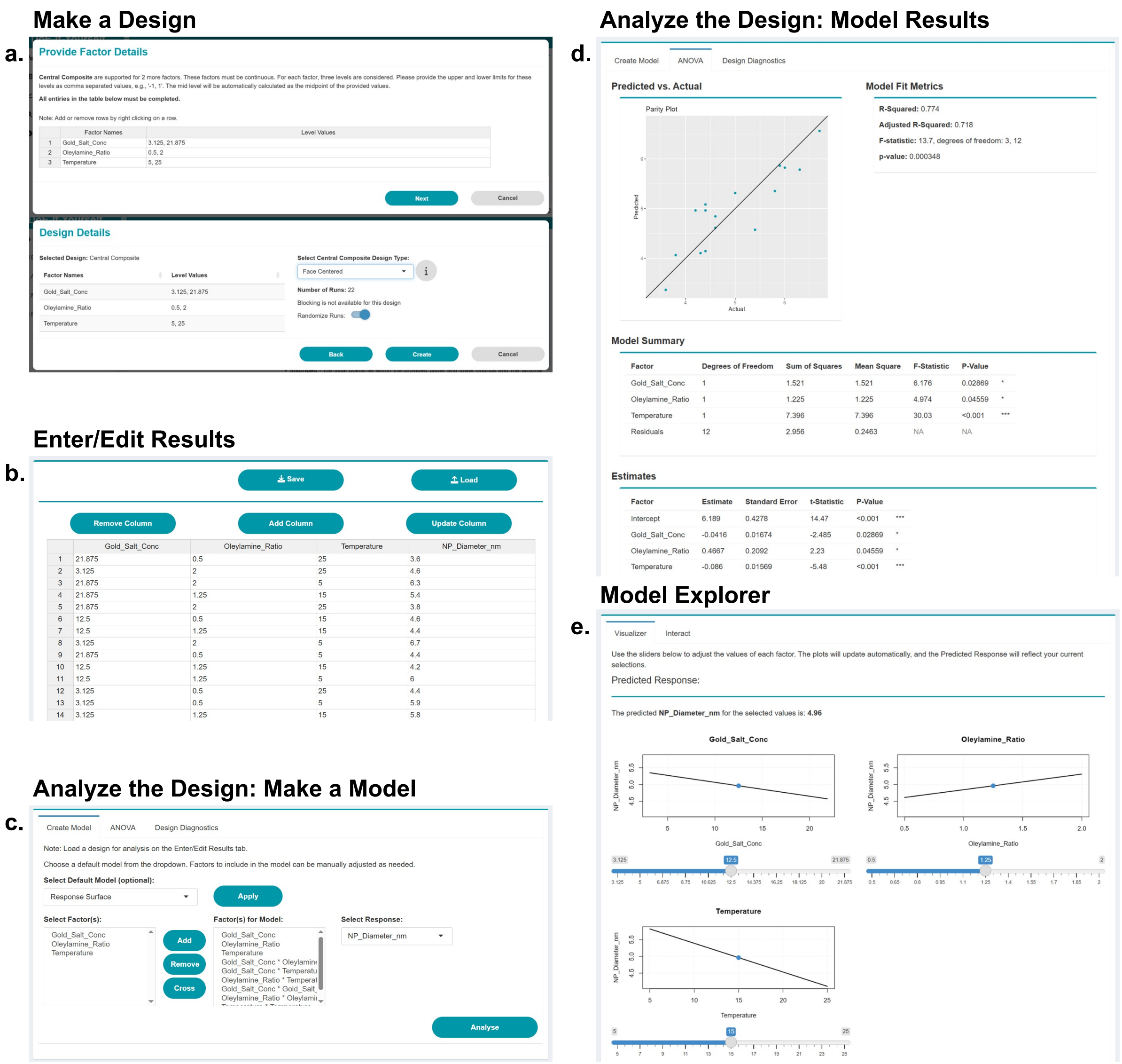

Figure S2: **Case Study 2: Central Composite design workflow.** Workflow for Case Study 2 demonstrating use of DoEIY. **a.** Creation of a face-centered Central Composite design using the **Make a Design** module. **b.** The design displayed in the **Enter/Edit Data** tab for entry of experimental results. **c.** Model specification and analysis using a response surface model within the **Analyze the Design** module. **d.** Model fitting results, including parity plot, goodness-of-fit statistics, ANOVA, and parameter estimates. **e.** Exploration of factor effects on the response using the **Model Explorer**.

Table S2: **Full experimental design for Case Study 2: Mean Nanoparticle Diameter** The inscribed, central composite design used to investigate the role of the gold salt concentration (mM), ratio of the capping agent to the reaction solvent, and the reaction temperature (°C) on the mean nanoparticle diameter.

**Run**	**Gold Salt Conc. (mM)**	**Ratio of Oleylamine to Reaction Solvent**	**Reaction Temp. (°C)**	**Diameter (nm)**	E	E_n
1	+1	-1	+1	3.6	1.48	0.41
2	-1	+1	+1	4.6	2.42	0.53
3	+1	+1	-1	6.3	1.63	0.26
4	+1	0	0	5.4	0.98	0.18
5	+1	+1	+1	3.8	2.02	0.54
6	0	-1	0	4.6	1.61	0.35
7	0	0	0	4.4	1.29	0.29
8	-1	+1	-1	6.7	3.97	0.59
9	+1	-1	-1	4.4	1.81	0.41
10	0	0	0	4.2	1.75	0.42
11	0	0	-1	6.0	1.55	0.26
12	-1	-1	+1	4.4	1.90	0.44
13	-1	-1	-1	5.9	1.55	0.26
14	-1	0	0	5.8	2.55	0.44
15	0	+1	0	5.0	1.64	0.33
16	0	0	+1	4.3	2.97	0.70
